# A Longitudinal Analysis of Cerebral Blood Flow in Perinatally HIV Infected Adolescents as Compared to Matched Healthy Controls

**DOI:** 10.3390/v13112179

**Published:** 2021-10-28

**Authors:** Jason G. van Genderen, Malon Van den Hof, Anne Marleen ter Haar, Charlotte Blokhuis, Vera C. Keil, Dasja Pajkrt, Henk J. M. M. Mutsaerts

**Affiliations:** 1 Location Academic Medical Center, Department of Pediatric Infectious Diseases, Emma Children’s Hospital, Amsterdam UMC, H8-253 P.O. Box 22660, 1100 DD Amsterdam, The Netherlands; m.vandenhof@amsterdamumc.nl (M.V.d.H.); a.m.terhaar@amsterdamumc.nl (A.M.t.H.); c.blokhuis@amsterdamumc.nl (C.B.); d.pajkrt@amsterdamumc.nl (D.P.); 2 Department of Radiology and Nuclear Medicine, Amsterdam UMC, Amsterdam Neuroscience, H8-253 P.O. Box 22660, 1100 DD Amsterdam, The Netherlands; v.c.w.keil@amsterdamumc.nl (V.C.K.); henkjanmutsaerts@gmail.com (H.J.M.M.M.)

**Keywords:** HIV, cerebral blood flow, cognitive function

## Abstract

Despite effective combination anti-retroviral therapy (cART), perinatally HIV infected (PHIV) adolescents still experience cognitive complications. We previously reported higher cerebral blood flow (CBF) in basal ganglia and white matter (WM) in PHIV children compared to matched controls. In healthy children CBF is associated with cognitive domains. To determine longitudinal changes in CBF and its impact on cognitive complications, we measured CBF—using arterial spin labeling—in 21 PHIV adolescents and 23 controls matched for age, sex and socio-economic status twice with a mean follow-up of 4.6 years. We explored associations between CBF changes and WM micro- and macrostructural markers and cognitive domains using linear mixed models. The median age at follow-up was comparable between PHIV adolescents 17.4y (IQR:15.3–20.7) and controls 16.2y (IQR:15.6–19.1). At baseline, PHIV had higher CBF in the caudate nucleus and putamen. CBF development was comparable in gray matter (GM), WM and subcortical regions in both groups. In our cohort, we found that over time an increase of GM CBF was associated with an increase of visual motor function (*p* = 0.043) and executive function (*p* = 0.045). Increase of CBF in the caudate nucleus, putamen and thalamus was associated with an increase processing speed (*p* = 0.033; 0.036; 0.003 respectively) and visual motor function (*p* = 0.023; 0.045; 0.003 respectively). CBF development is relatively normal in PHIV adolescents on cART. CBF decline is associated with cognitive impairment, irrespective of HIV status.

## 1. Introduction

The introduction of effective combination antiretroviral therapy (cART) has led to an increase of the life expectancy of perinatally human immunodeficiency virus (HIV)-infected (PHIV) adolescents [1]. Despite these effective therapies, there is growing evidence suggesting that PHIV children and adolescents have an altered brain structure and function compared to HIV uninfected peers [2].

Lesions in brain white matter (WM)—i.e., WM hyperintensities (WMH)—are thought to be of vascular origin [3]. In PHIV children, functional brain changes include altered cerebral blood flow (CBF) compared to matched controls, suggesting possible vascular disease [4]. Moreover, PHIV children exhibited different neurometabolite concentrations compared to controls, suggestive of glial proliferation, and these findings were associated with poorer cognitive performance [5,6]. Some cross-sectional studies report a higher prevalence of white matter (WM) macro- and microstructural damage—i.e., WMH and increased white matter diffusivity—in PHIV children and adolescents compared to (matched) controls [7,8,9,10,11], although this could not consistently be replicated in a longitudinal study [12]. Subcortical regions may also be affected, as reduced volumes of caudate nucleus and putamen have been repeatedly associated with cognitive impairment in HIV-infected adults [13]. In both treated and untreated PHIV children, cerebral blood flow alterations and calcifications have been reported in those regions [14]. Finally, differences in cognitive function, including decrease in executive function and lower intelligence quotient (IQ), have been reported in PHIV children and adolescents compared to controls [15,16,17].

The role of CBF in relation to structural and other functional brain changes in PHIV children and adolescents is rarely investigated [2] and incompletely understood, despite CBF being a pivotal parameter for neuronal function [18] and in light of possible HIV associated vasculopathy [19]. Our previous cross-sectional assessment—the only study assessing CBF in treated PHIV children—found lower CBF in gray matter (GM) associated with a higher prevalence of WM lesions in PHIV children compared to matched controls [4]. In HIV infected adults, studies reported decreased CBF compared to controls, which could reflect accelerated vascular ageing [20,21].

Arterial spin labeling (ASL) facilitates studying CBF in children, as it is a noninvasive magnetic resonance imaging (MRI) technique that measures CBF by using arterial blood water as endogenous tracer [22]. ASL has been used to estimate the association between CBF and cognition in other pediatric populations [23,24].

To gain further insight in the pathophysiological mechanisms of HIV associated cognitive complications and clinical consequences in PHIV adolescents, we longitudinally determined changes in CBF in GM, WM and subcortical GM structures. We explored possible relations with other structural and functional brain MRI biomarkers, including WM damage and cognitive function over time.

## 2. Materials and Methods

### 2.1. Study Design and Participants

This study is part of the Neurological, cOgnitive and VIsual performance in perinatally HIV infected childrEn (NOVICE) study: a cohort study investigating the neurological, cognitive and ophthalmological outcomes of PHIV adolescents compared to controls matched for age, sex, socio-economic status and ethnic background [25]. In our previous analysis, we included 35 PHIV children and adolescents aged between 8 and 18 years visiting our outpatient clinic, and 37 matched controls from similar communities and socio-economic status between December 2012 and January 2014 [4]. Between February 2017 and July 2018 all participants were approached to participate for follow-up. The following exclusion criteria were used: current or past neurological or psychiatric disorders not associated with HIV, a history of traumatic brain injury resulting a loss of consciousness of more than 30 min, intracerebral neoplasms and MRI contraindications including metal implants or claustrophobia; the complete criteria for non-inclusion have been previously published in detail [12]. The ethics committee of the Academic Medical Center (AMC), part of the Amsterdam University Medical Centers, approved the study protocol. We adhered to the tenets of the Declaration of Helsinki and obtained written informed consent from all participants older than 12 years and from participants’ parents younger than 18 years of age. The NOVICE cohort study is registered at the Dutch Trial registry (identifier NL6813).

### 2.2. MRI Data Acquisition

We performed magnetic resonance imaging (MRI) scans of participants who provided informed consent for follow-up. The MRI scans were performed with the same 3T MRI scanner (Ingenia, Philips, Best, The Netherlands) equipped with the same 16-channel phase array head coil. The NOVICE MRI protocol included a 3D T1-weighted sequence for volumetry and diffusion tensor imaging (DTI) for WM microstructure and 3D fluid attenuated inversion recovery (FLAIR) for WMH. The MRI protocol parameters for these scans have been described in detail previously [12].

CBF maps were obtained using 2D EPI pseudo-continuous ASL (pCASL) with 20 axial 6 mm slices with 0.6 mm slice gap and a 240 × 240 mm^2^ field of view resulting in a 3 × 3 × 6.6 mm^3^ resolution, labeling duration = 1650 milliseconds (ms), and 30 control-label pairs. Due to a clinical scanner software upgrade, there was a minimal change in the shortest repetition time, with echo time/repetition time (TR) = 14/4000 ms at baseline and 16/4000 ms at follow-up, and post labeling delay ranging from the inferior to superior slice 1525–2275 ms at baseline and 1525–2340 ms at follow-up

### 2.3. Image Processing

**Figure 1:**
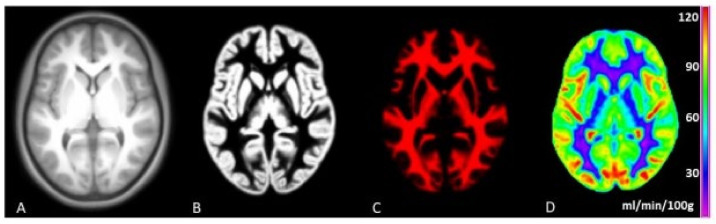
ExploreASL workflow This figure demonstrates several stages of the ExploreASL work flow to calculate a cerebral blood flow (CBF) map. (**A**) = a structural 3D T1 scan. (**B**) = a segmented gray matter map. (**C**) = segmented white matter map. (**D**) = a CBF map.

We used ExploreASL (version 1.2.0) for the processing of 3D T1w, FLAIR and ASL images [26]. Figure 1 shows part of the ExploreASL work flow. Scans of insufficient quality due to excessive head motion or artefacts were excluded (by J.G.v.G. and H.J.M.M.M, the latter having eight years of ASL experience) and after consulting an experienced neuroradiologist (V.C.K.) in case of doubt. We chose the same regions of interest as in our previous study: GM, WM, caudate nucleus, putamen and thalamus, as cerebral injury in PHIV children mostly occurs in GM, WM and subcortical regions, and damage in the subcortical regions is associated with cognitive impairment [14,27].

There were no statistically significant hematocrit differences, adjusted for age and sex, between PHIV adolescents and controls (*p* = *0*.496); hence we assumed the same blood T1 value for all participants.

Due to an MRI scanner software update in the years between baseline and follow-up, the scanning protocols differed slightly. To account for these instrumental differences, we used individual CBF ratios with the assumption that changes in the control group over time are due to these instrumental changes. We calculated CBF ratios by dividing all individual CBF values (in mL/100 g/min) by the mean GM CBF of controls (in mL/100 g/min), separately for baseline and follow-up each. We described the image processing of the FLAIR and DTI scans previously in detail [12].

### 2.4. Demographic and HIV Related Variables

Historical cART- and HIV related characteristics of the PHIV participants were collected from patient records or provided by the Dutch HIV monitoring foundation [28]. We defined cART as the use of at least three antiretroviral drugs from a minimum of two drug classes. In all controls an HIV negative status was re-confirmed as reported previously [25].

### 2.5. Cognitive Functioning

Neuropsychological assessments were performed by a well-trained neuropsychologist (A.M.t.H) who was unaware of the participants’ HIV status. We used a test battery identical to the first visit to assess intelligence quotient (IQ) and various cognitive domains including processing speed, learning ability, visual-motor function and executive function [25].

### 2.6. Statistical Analyses

We performed statistical analyses using R version 3.5.1 (R Core team, Vienna, Austria) [29]. We considered *p* < 0.05 as statistically significant. We compared demographic variables between PHIV adolescents and controls using the Mann–Whitney *U* test (non-normally distributed) or Students’ *t* test (normally distributed) for continuous data, and Fisher’s exact test for categorical data. We used linear mixed models to assess development or changes in CBF in GM, WM, caudate nucleus, putamen and thalamus over time, while adjusting for age and sex. In PHIV adolescents, we assessed associations between changes in CBF and HIV related characteristics. For PHIV and controls, we also assessed associations between CBF and WMH volume and diffusion parameters: fractional anisotropy (FA), mean diffusivity (MD), axial diffusivity (AD) and radial diffusivity (RD) over time. Finally, we assessed associations between CBF and the following cognitive domains: IQ, processing speed, learning ability, visual motor function and executive function; while adjusting for age, sex, and IQ (in all domains except IQ), in PHIV and controls. We did not adjust for hematocrit in both groups due to comparable hematocrit blood values. We did not apply multiple comparison adjustments due to the explorative character of the study.

## 3. Results

The number of participants who consented to MRI for follow-up was similar between groups: 21/34 (62%) PHIV and 23/37 (62%) controls. For the study dropouts, reasons to not participate were: disinclination to participate (9 in both groups), inability to contact (5 controls) or relocation (2 PHIV). The follow-up participants did not differ from the dropout participants in baseline GM or WM CBF (all *p* > 0.05). We excluded 1 PHIV and 3 controls from MRI analyses due to poor ASL scan quality caused by head motion.

The median age (years) at the second enrollment was similar between PHIV adolescents (17.4 (IQR: 15.3–20.7)) and controls (16.2 (IQR: 15.6–19.1)). There were no differences in participants’ characteristics between PHIV adolescents and controls (Table 1).

Of the participating PHIV adolescents, 19 (90%) had an undetectable HIV viral load (VL) at the follow-up, and 15 (71%) PHIV adolescents maintained an undetectable HIV VL during the entire follow-up period. Two PHIV adolescents had detectable HIV VLs of 1515 and 32,996 copies/mL. The median age at cART initiation was 2.5 years (IQR: 1.2–4.3).

### 3.1. Differences in CBF between Baseline and Follow-Up

**Figure 2:**
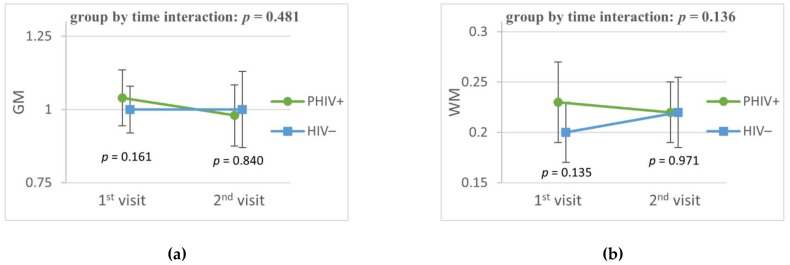

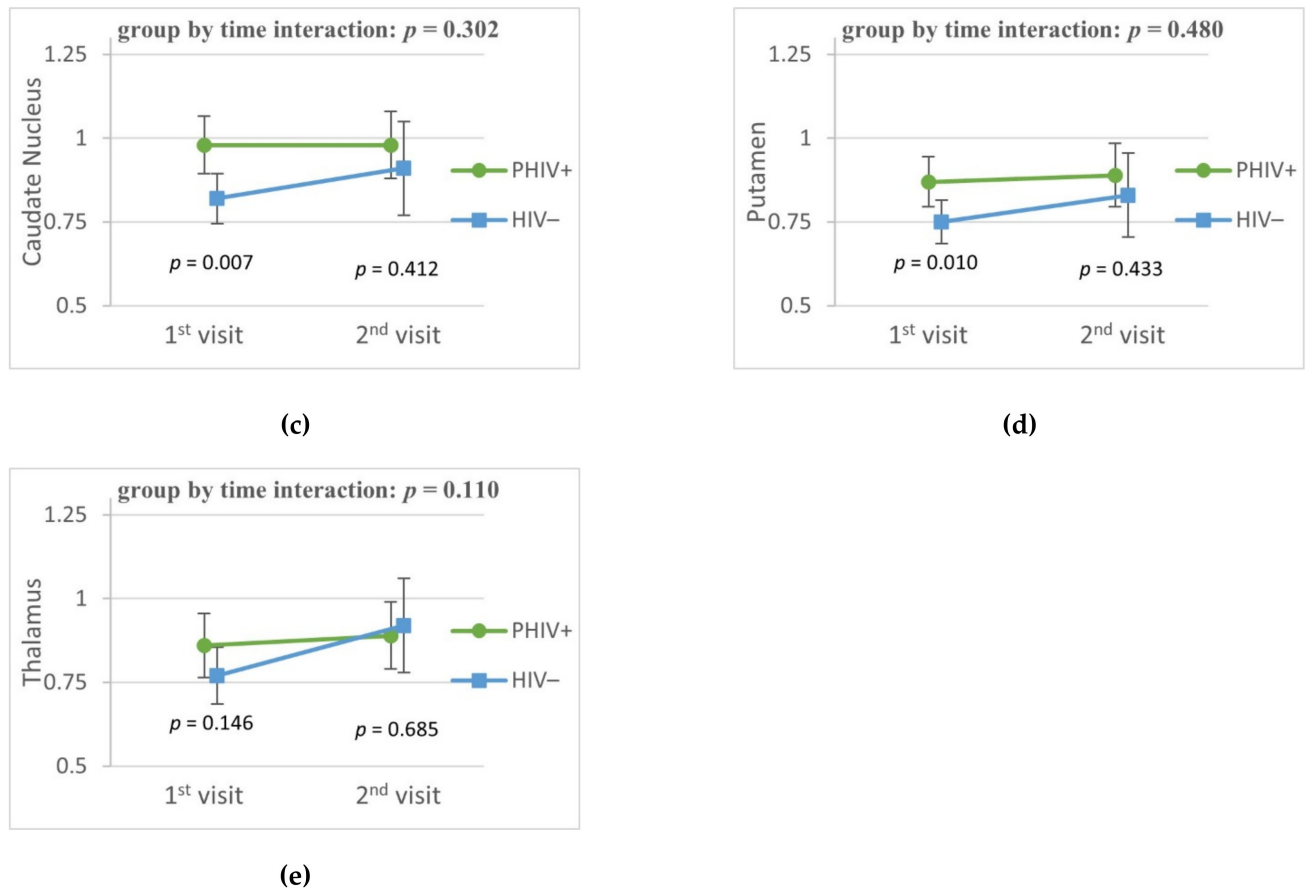
CBF changes in cohort. The graphs show longitudinal changes in CBF of PHIV+ adolescents compared to controls matched for age, sex, ethnic background. Figures show plotted least square means and their 95% confidence interval of CBF in the specific region as a ratio to mean GM CBF of controls. (**a**) = gray matter; (**b**) = white matter; (**c**) = caudate nucleus; (**d**) = putamen and (**e**) = thalamus. *P* values are the group by time interaction adjusted for age and sex. Additional *p* values are the cross-sectional difference at first and second visit. Abbreviation: CBF = cerebral blood flow; GM = gray matter, WM = white matter.

At baseline, PHIV adolescents had higher CBF in the caudate nucleus (*p* = 0.007) and putamen (*p* = 0.010) compared to matched controls. At follow-up, the CBF in the caudate nucleus and putamen did not differ between PHIV and controls. CBF in GM, WM and in the thalamus were comparable between groups at both baseline and follow-up (Figure 2). We found similar results using individual hematocrit adjusted blood T1 values (data not shown). In PHIV adolescents we found no difference in CBF in a sensitivity analysis comparing those with a detectable VL during follow-up and those who maintained an undetectable VL (data not shown).

### 3.2. Determinants of Changes in CBF

We found that a decline in WM CBF was associated with a decline of AD, one of four parameters indicating WM microstructure (Table 2). We also found that increase in GM CBF was associated with an increase of visual motor function and executive function. Increase of caudate nucleus, putamen and thalamus CBF was associated with increase of processing speed and visual motor function (Table 3). We found no associations between changes in CBF and HIV associated characteristics (Supplementary Table S1).

## 4. Discussion

This study has three main findings. Firstly, the at baseline increased caudate nucleus and putamen CBF in PHIV was normalized at follow-up. Secondly, we found an association between WM CBF and WM microstructural integrity. Finally, CBF was associated with several cognitive domains, including processing speed, visual motor function and executive function.

In healthy individuals, CBF physiologically declines with age and during adolescence CBF rapidly declines in different brain areas [30]. This could be an explanation of the observed difference at baseline of CBF in the caudate nucleus and thalamus in PHIV children and controls as participants might undergo these physiological changes at different moments. A higher CBF in subcortical regions could also have reflected hypermetabolism, which might be linked to biphasic psychomotor disease reported in adults [31]. The lack of clinical symptoms and CBF differences between PHIV adolescents and controls at follow-up reject this hypothesis. With our findings, we hypothesized that normalization of CBF between PHIV adolescents and controls could be due to effective viral suppression (preventing vasculopathy) [19] and/or physiological plasticity of the brain (and its blood vessels).

In HIV infected adults compared to controls, lower CBF was reported in the neocortex or GM [20,21], which suggests an acceleration of physiological CBF decline later in life. To gain insight in the possible decrease in GM CBF, it would be of interest to monitor PHIV adolescents as they age, and compare them to adults who acquired HIV later in life.

We did not find an association between CBF and WMH volume. It is hypothesized that the origin of WMH is-at least partially-vascular [3]. Despite analyzing the total WM CBF, the relative low reliability of ASL could have hampered the detection of such association, as subtle focal WM CBF changes are not detected.

We previously reported that WM microstructural differences diminished in PHIV adolescents and controls over time [12]. Nonetheless, evidence suggests a higher prevalence of cerebral injury, including WM microstructural damage, in PHIV adolescents compared to controls [2]. In this study, we found that decline in WM CBF is associated with a decline in AD, reflecting axonal injury—which may demonstrate a vascular component in microstructural WM damage.

The associations between GM CBF and multiple cognitive domains are in line with previously reported results investigating other pediatric populations [23,24]. Evidence suggest that perinatal HIV infection is associated with reduced executive function [17]. Although we cannot differentiate causal effects with these data, we hypothesized that subtle differences in GM CBF are markers of early microscopic brain damage in PHIV adolescents, which in turn raises susceptibility to impaired cognitive performance.

Our longitudinal study investigated brain complications in PHIV adolescents as a proxy for disease progression by using advanced noninvasive MRI technique. Our findings contribute to the further understanding of the development of long-term complications of a perinatal HIV infection. As these PHIV patients are reaching adulthood, it is pivotal to understand how their perinatal HIV infection evolves during adolescence while under optimal cART.

Our study has a few potential limitations. Our study is a single center study, and the small numbers of participants might have precluded us from detecting subtle associations and reduced the generalizability. We did not perform large multivariate analyses or subgroup analyses in PHIV adolescents to avoid overfitting of the statistical models [32]. This precluded us from assessing changes in CBF to MRI markers or cognitive profiles in PHIV adolescents. The MRI scanner software upgrade might have caused differences in the observations, although this affects all participants equally and we were able to mitigate this using CBF ratios with healthy controls who were matched by age and sex to PHIV adolescents.

## 5. Conclusions

Despite initial differences at baseline, adequately cART-treated PHIV adolescents have relatively normal CBF evolution. CBF decline in growing adolescents is associated with WM microstructural changes and impairments in specific cognitive domains. These findings warrant future large international multicenter trials, that may identify differences in CBF and in association with cognitive development and its consequences in PHIV adolescents.

## Figures and Tables

**Table 1:** Participants’ characteristics.

	PHIV (*n* = 21)	CONTROLS (*n* = 23)	*p*
FU rate	62%	62%	
FU time (years)	4.60 (0.34)	4.60 (0.34)	0.694 ^X^
Age at baseline (years)	13.1 (10.8–15.7)	11.6 (11.0–14.4)	0.181 ^Z^
Age at follow-up (years)	17.4 (15.3–20.7)	16.2 (15.6–19.1)	0.441 ^Z^
Male sex	12 (57%)	9 (40%)	0.197 ^Y^
Ethnic background			
Black	15 (75%)	13 (65%)	0.731 ^Y^
Hematocrit (l/l)	0.43 (0.40–0.45)	0.40 (0.39–0.44)	0.496 ^Z^
Blood pressure (mmHg)			
Systolic	123 (115–132)	120 (113–124)	0.600 ^Z^
Diastolic	66 (59–74)	64 (60–73)	0.928 ^Z^
MRI scan of good quality	20 (95%)	20 (87%)	
Mean motion (mm)	0.13 (0.10–0.18)	0.13 (0.11–0.18)	0.301 ^Z^
GM/ICV ratio	0.50 (0.03)	0.49 (0.02)	0.207 ^X^
WM/ICV ratio	0.34 (0.02)	0.35 (0.02)	0.686 ^X^
WMH volume (mm^3^)	90 (17–163)	42 (20–72)	0.355 ^Z^
Age at HIV diagnosis (years)	1.5 (0.8–4.1)		
CDC category			
NA	8 (40%)		
B	7 (35%)		
C	5 (25%)		
Undetectable viral load	18 (90%)		
Undetectable entire follow-up	14 (70%)		
HIV viral load zenith (ln)	12.8 (11.5–13.4)		
CD4^+^ T-cell nadir *Z* score	−0.83 (0.63)		
Age cART initiation	2.5 (1.2–4.3)		
Current cART use	19 (95%)		

Values are noted in number and percentage, mean and standard deviation or median and inter-quartile range. CD4^+^ T-cell nadir *Z* score is age-adjusted. Statistical tests: X = Student’s *t* test; Y = Fisher’s exact test; Z = Mann–Whitney U test. Abbreviations: CDC = Center for Disease Control and Prevention, NA = no to minimal symptoms to AIDS, B = moderate symptoms, C = severe symptoms or AIDS; FU = follow up; HIV = human immunodeficiency virus; IQR = inter-quartile range; kg = kilogram; l = liter; m = meter; mm = millimeter; n = amount; SD = standard deviation; y = years. Deviation in number (PHIV): height (19); weight (19); BMI (19); systolic (19); diastolic (19); treatment initiation and duration (18); CD4^+^ T-cell nadir (19) HIV zenith (18). Deviation in number controls: systolic (18); diastolic (18).

**Table 2:** Association between changes in CBF and WM damage markers in entire cohort.

	FA		MD		AD		RD		WMH Volume	
	coefficient (95%CI)	*p*	coefficient (95%CI)	*p*	coefficient (95%CI)	*p*	coefficient (95%CI)	*p*	coefficient (95%CI)	*p*
GM	−0.177 (−0.99 to 0.63)	0.672	0.01 (−0.01 to 0.03)	0.240	0.02 (−0.002 to 0.05)	0.095	0.005 (−0.01 to 0.03)	0.647	0.17 (−0.53 to 0.88)	0.639
WM	−0.79 (−3.3 to 1.69)	0.542	0.06 (0.0003 to 0.11)	0.062	0.09 (0.02 to 0.17)	0.027	0.04 (−0.02 to 0.09)	0.199	−0.06 (−1.92 to 1.83)	0.947

Linear mixed models to assess associations between white matter damage markers and cerebral blood flow in GM and WM. Models adjusted for age, sex and HIV status. Abbreviations: AD = axial diffusivity; CI = confidence interval; FA = fractional anisotropy; GM = gray matter; MD = mean diffusivity; RD = radial diffusivity; WM = white matter. Parameters have been multiplied by a factor for interpretation purposes due to low values. FA (×100) MD/AD/RD (×1000).

**Table 3:** Association between CBF and cognitive domains in entire cohort.

	IQ		Processing Speed		Learning Ability		Visual Motor Function		Executive Function	
	coefficient (95%CI)	*p*	coefficient (95%CI)	*p*	coefficient (95%CI)	*p*	coefficient (95%CI)	*p*	coefficient (95%CI)	*p*
GM	−1.63 (−11 to 8.8)	0.751	13.0 (−0.78 to 27)	0.078	−9.3 (−30 to 11)	0.390	9.18 (0.84 to 18)	0.042	1.36 (−0.50 to 2.59)	0.045
WM	−2.0 (−4.9 to 0.98)	0.195	1.86 (−2.3 to 6.0)	0.397	−3.3 (−9.1 to 2.6)	0.295	1.41 (−1.1 to 4.1)	0.296	0.34 (−0.16 to 0.70)	0.121
Caudate Nucleus	−5.65 (−15 to 4.01)	0.238	14.9 (1.90 to 28)	0.033	−11 (−30 to 8.8)	0.303	9.76 (1.85 to 18.5)	0.023	0.47 (−1.14 to 1.51)	0.486
Putamen	−2.88 (−13 to 7.61)	0.582	16.1 (1.88 to 31)	0.036	−6.6 (−28 to 15)	0.562	9.55 (0.61 to 20)	0.045	0.18 (−1.49 to 1.43)	0.806
Thalamus	−1.95 (−12 to 8.2)	0.701	20 (7.8 to 33)	0.003	−2.15 (−21 to 16)	0.826	12.5 (4.9 to 21)	0.003	0.74 (−0.84 to 1.77)	0.265

Linear mixed models to assess associations between cognitive domains and cerebral blood flow in GM and WM. All models were adjusted for age, sex, HIV status and IQ. The model assessing IQ was not additionally adjusted for IQ. Abbreviations: CI = confidence interval; GM = gray matter; IQ = intelligent quotient; WM = white matter.

## Data Availability

Data cannot be shared publicly because the data contain (potentially) sensitive patient information. Data are available (in anonymous form) upon request to the corresponding author.

## Supplementary Materials

The following are available online at https://www.mdpi.com/article/10.3390/v13112179/s1: Associations between CBF changes and HIV associated characteristics.
