# Supplementary material for: A Longitudinal Analysis of Cerebral Blood Flow in Perinatally HIV Infected Adolescents as Compared to Matched Healthy Controls
Source: Viruses. 2021 Oct 28;13(11):2179. doi: 10.3390/v13112179 (PMC8625391; doi:10.3390/v13112179)
Supplement: Supplementary file 1 [file viruses-13-02179-s001.zip › viruses-1396685-supplementary.pdf]

Supplementary Materials

Table S1. Associations between CBF and HIV associated characteristics

	GM		WM		Caudate nucleus		Putamen		Thalamus	
	coefficient (95%CI)	*p*	coefficient (95%CI)	*p*	coefficient (95%CI)	*p*	coefficient (95%CI)	*p*	coefficient (95%CI)	*p*
VL Zenith	−0.02 (−0.1 to 0.05)	0.610	−0.009 (−0.03 to 0.012)	0.441	−0.02 (−0.09 to 0.05)	0.996	−0.03 (−0.09 to 0.04)	0.499	−0.01 (−0.09 to 0.06)	0.772
CDC B	0.04 (−0.16 to 0.23)	0.734	−0.05 (−0.10 to 0.01)	0.175	−0.012 (−0.21 to 0.18)	0.915	−0.002 (−0.18 to 0.18)	0.985	0.14 (−0.05 to 0.33)	0.219
CDC C	−0.01 (−0.23 to .20)	0.911	−0.02 (−0.08 to 0.05)	0.657	0.003 (−0.21 to 0.21)	0.980	−0.008 (−0.21 to 0.19)	0.945	0.13 (−0.07 to 0.34)	0.271
$CD4^{+}$ T-cell *Z* score	−0.08 (−0.14 to 0.12)	0.916	0.008 (−0.03 to 0.04)	0.703	0.02 (−0.10 to 0.15)	0.749	0.004 (−0.12 to 0.12)	0.952	−0.03 (−0.17 to 0.11)	0.703
Age cART initiation	−0.01 (−0.04 to 0.01)	0.326	−0.002 (-0.008 to 0.005)	0.639	−0.01 (−0.04 to 0.006)	0.213	−0.01 (−0.03 to 0.01)	0.396	−0.01 (−0.04 to 0.01)	0.417
Undetectable VL*	0.13 (−0.02 to 0.30)	0.116	0.02 (−0.03 to 0.08)	0.456	0.15 (−0.002 to 0.31)	0.081	0.14 (−0.008 to 0.28)	0.096	0.14 (−0.02 to 0.31)	0.122

Linear mixed models to assess associations between changes in CBF and (historical) HIV-related parameters. Models adjusted for age and sex. *Undetectable during entire follow-up. Abbreviations: cART = combination antiretroviral therapy; CDC = center for disease prevention and control, B = moderate symptoms, C = severe symptoms or AIDS; CI = confidence interval; GM = gray matter; VL = viral load (logarithmically transformed); WM = white matter; WMH = white matter hyperintensities
